# The Impact of Media Reporting on the Emergence of Charcoal Burning Suicide in Taiwan

**DOI:** 10.1371/journal.pone.0055000

**Published:** 2013-01-30

**Authors:** Ying-Yeh Chen, Feng Chen, David Gunnell, Paul S. F. Yip

**Affiliations:** 1 Taipei City Psychiatric Center, Taipei City Hospital, Taipei City, Taiwan; 2 Institute of Public Health and Department of Public Health, National Yang-Ming University, Taipei City, Taiwan; 3 Department of Statistics, University of New South Wales, Sydney, Australia; 4 School of Social and Community Medicine, University of Bristol, Bristol, United Kingdom; 5 Hong Kong Jockey Club Center for Suicide Research and Prevention, University of Hong Kong, Hong Kong, China; 6 Department of Social Work and Social Administration, University of Hong Kong, Hong Kong, China; University of Warwick, United Kingdom

## Abstract

We investigated the association of the intensity of newspaper reporting of charcoal burning suicide with the incidence of such deaths in Taiwan during 1998–2002. A counting process approach was used to estimate the incidence of suicides and intensity of news reporting. Conditional Poisson generalized linear autoregressive models were performed to assess the association of the intensity of newspaper reporting of charcoal burning and non-charcoal burning suicides with the actual number of charcoal burning and non-charcoal burning suicides the following day. We found that increases in the reporting of charcoal burning suicide were associated with increases in the incidence of charcoal burning suicide on the following day, with each reported charcoal burning news item being associated with a 16% increase in next day charcoal burning suicide (p<.0001). However, the reporting of other methods of suicide was not related to their incidence. We conclude that extensive media reporting of charcoal burning suicides appears to have contributed to the rapid rise in the incidence of the novel method in Taiwan during the initial stage of the suicide epidemic. Regulating media reporting of novel suicide methods may prevent an epidemic spread of such new methods.

## Introduction

A rapid increase in suicides by burning barbecue charcoal changed the epidemiology of suicide in Taiwan in the first decade of the 21^st^ century [1], [2]. The incidence of charcoal burning suicide increased from 0.1 to 5.1 per 100,000 population between 1998 and 2010, making it the second most common method of suicide (after hanging) in Taiwan, accounting for more than 30% of suicides in 2010 [3].

Previous reports have suggested that media glamorization of this novel method was responsible for the dramatic increase in charcoal burning suicide [4], [5]. The first widely-reported case of charcoal burning suicide occurred in Hong Kong in 1998 where a middle-aged woman was depicted as having a peaceful and painless death by burning barbecue charcoal in a small sealed room [4], [6]. It is believed the method was subsequently adopted in Taiwan because the same written language (i.e. traditional Chinese characters) is used in both countries, thus reports of its use were accessible to the whole population. The shared cultural belief on the importance of maintaining the appearance of the body for a better next life may also contribute to its spread in these two regions. An interview-based study in Taiwan found that 87% of individuals who survived a suicide attempt where charcoal burning was used said that media reporting contributed to their choice of this method [5]. However, no previous studies have systematically examined the intensity of reporting of this new method when it first appeared in Taiwan and whether the intensity of media reporting was associated with the increasing incidence of suicides by charcoal burning.

The aim of the current analysis is to explore the role of the newspaper reporting in the dissemination of charcoal burning suicide in Taiwan during 1998–2002. We assessed whether the intensity of newspaper reporting was associated with subsequent increases in charcoal burning suicides.

## Methods

### Ethics Statement

Because no personal data were involved, this study was exempted from ethical review by the Human Research Ethics Committee, Taipei City Hospital, Taiwan.

### Data

During 1998–2002, three major newspapers, China Times (CT), United Daily (UD) and Liberty Times (LT) accounted for more than 90% of newspaper sales in Taiwan with approximately equal market shares for these three newspapers [7]. The content of two (UD and CT) of these three papers from the late 1990s/early 2000s was available in electronic archives. Using online search tools, the United Daily News Dataset (for UD), and Knowledge Media Winner (for CT), were searched for news articles containing Chinese key words related to suicide and suicide method (see appendix) published between1998–2002 (N = 9675 for UN; N = 11468 for CT). Editorials commentaries, fictional stories, suicide terrorism, and articles providing general information about suicide prevention were excluded. A total of 1898 and 3689 suicide news items for UD and CT were retrieved respectively. Articles were then read individually to identify those that reported charcoal burning suicide vs. other methods of suicide. Altogether 176 and 158 news items for UD and CT respectively on charcoal burning suicide and 1722 (UD) and 3531 (CT) on other methods of suicide were identified.

The electronic archives for LT did not cover the study period. To assess possible bias from the exclusion of this paper, we randomly selected two months in 2001/2002 and hand searched the newspaper archives to investigate the concordance of suicide reporting in LT with UD and CT. The reporting of charcoal burning suicides in the LT was lower than the other two daily newspapers. In the two months selected in 2001, we identified a total of ten news items on charcoal burning suicide in UD, seven in CT and only one news item in LT. In 2002, 23 news items on charcoal burning suicide were identified for UD and 19 for CT, but only 3 news items on charcoal burning suicide were reported in LT, indicating the reporting of charcoal burning suicide in LT was probably lower than in the other two newspapers. Throughout the study period, suicide news stories (including those of charcoal burning suicides) were never reported on the front pages of the papers reviewed.

Data on suicide deaths, classified according to the International Classification of Disease (ICD-9) were obtained from official death records in Taiwan. The ICD-9 codes used were deaths registered in E950–959 (intentional self-harm) and E980–989 (intent undetermined). Deaths certified as undetermined intent were also included because previous research indicated that many suicide deaths were included in this category [8]. There is no specific ICD-9 code for charcoal-burning suicide. Such deaths are coded as E952/E982 (suicide or undetermined death by poisoning using nondomestic gas). Before 1998, E952/E982 accounted for less than two percent of suicide deaths in Taiwan. Hence, we assumed that most of the suicides coded E952/E982 subsequent to 1998 were by charcoal burning [9]. Therefore, E952/E982 was used to denote charcoal-burning suicide in our analysis.

### Analytic Strategy

The cumulative number of charcoal burning (or non-charcoal burning) news items about suicide from time 0 (beginning of 1998) up to time 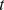 was modeled by a counting process 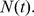 The intensity process 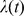 of 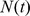 is assumed to be
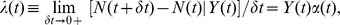
where 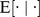 denotes conditional expectation [10]–[13]. In this model the exposure process 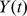 represents the number of charcoal burning (or non-charcoal burning) suicide cases on the day before *t*, and the intensity function 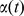 represents the ratio of the expected daily number of reports to the number of charcoal burning (or non-charcoal burning). The time-varying reporting intensity 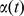 and its first order derivative 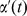 were then estimated using a nonparametric curve estimation method, the local polynomial method [10]–[12]. The crude charcoal burning (or non-charcoal burning) incidence rate in cases per day ( = intensity), and its first order derivative – a measure of the rate of change in incidence – were also estimated using this method with 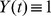. These estimated incidences and rates of change in incidence (first order derivatives) curves were then plotted against aligned calendar time (in days) to illustrate the relationship between suicide reporting and suicide incidence graphically.

To formally assess the potential impact of news reporting of charcoal burning suicides on suicide incidence, analyses using conditional Poisson generalized linear autoregressive models were performed. We first fitted an unadjusted Poisson generalized linear autoregressive model to examine the effect of newspaper reporting of charcoal burning suicide on the incidence of charcoal burning deaths. In this model only lag terms of the predictor (i.e. daily number of newspaper reports of charcoal burning suicide) and outcome (i.e. daily count of charcoal burning suicide) were included. We then fitted an adjusted model by adding potential confounders to the model. The potential confounders were the daily count of non-charcoal burning suicide (which served as an indicator of background suicide rate) and day of the week (as the number of newspaper pages and suicide count could vary according to different days of the week). Equivalent models for the impact of news reporting of non-charcoal burning suicide on non-charcoal burning suicide deaths were fitted as well. Specifically, the Poisson generalized linear autoregressive model we used for the count of charcoal burning suicides 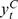 is
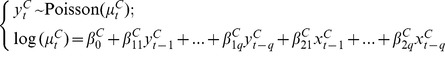
(1)where 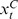 denotes the amount of newspaper reports of charcoal burning suicides on day *t*. The same approach was used for suicides by methods other than charcoal burning. The adjusted models are obtained by including *q* lagged terms (up to one week) of the corresponding covariates. Our primary hypothesis concerned the impact of news reporting on the incidence of charcoal burning suicides, but of course if there were no suicides there would be no reporting, so we also investigated the strength of association between the incidence of suicides and subsequent reporting of these deaths. To do this we used a Poisson generalized linear autoregressive model similar to (1) except that the number of reported news items acted as the dependent variable. For interpretability, the lag parameter *q* was set to 7 in all the aforementioned models as prior studies tended to assume that the effect of news reporting of non-celebrity suicides lasted for about 1 week [14], [15].

## Results

**Figure 1:**
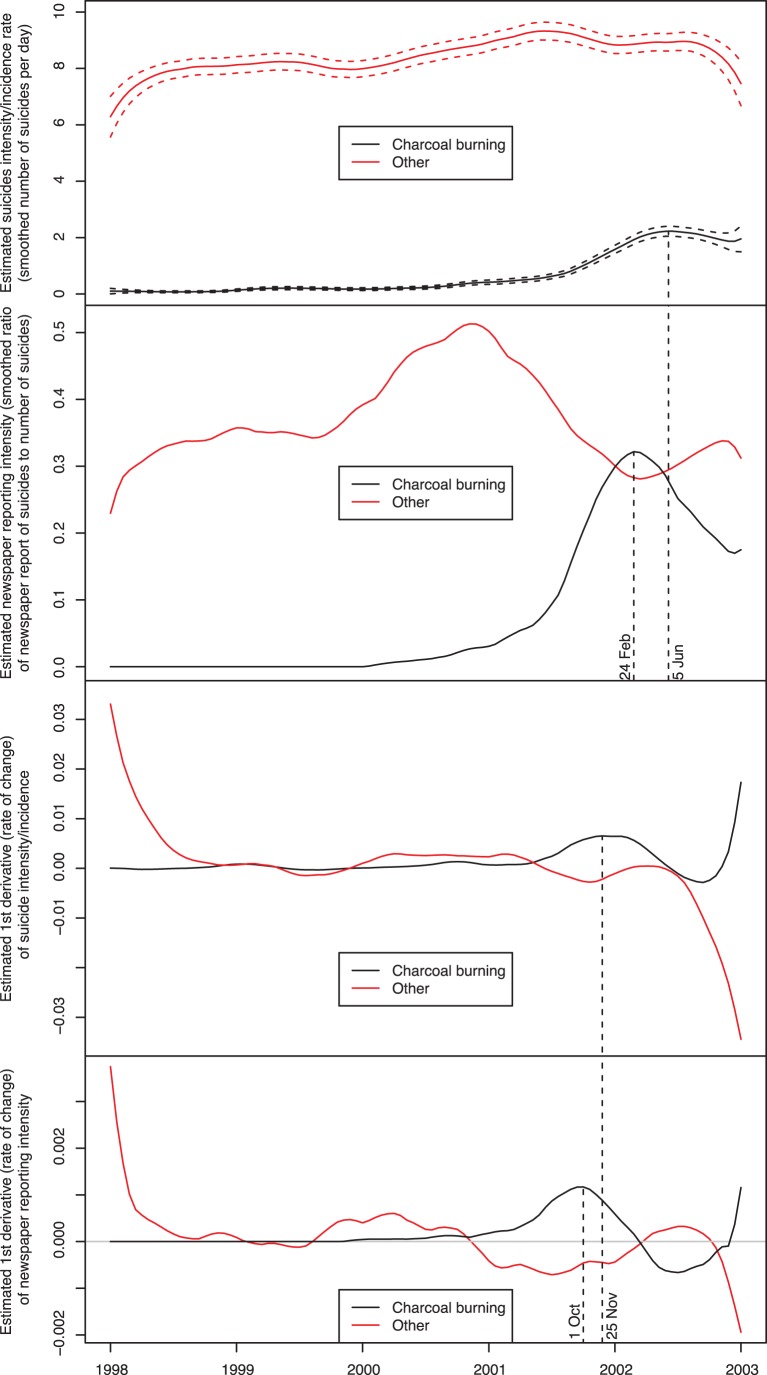
The relationships between newspaper reporting intensity of charcoal burning suicides and suicide incidence in Taiwan, 1998–2002.

The incidence of charcoal burning suicide began to increase in 2000, with a more rapid and prominent rise in 2001, whereas the incidence of suicide using all other methods was relatively stable during the study period with a slight fall towards the end of 2002 (top panel, Figure 1) indicating some degree of method substitution. Newspaper reporting intensity of charcoal burning suicide (the number of reports per death) started to rise after 2000, a sharp increase occurred midway through 2001 and reporting peaked on 24th Feb. 2002 and subsequently declined (second panel, Figure 1). The reporting intensity for other methods of suicide was higher than charcoal burning suicide before the end of 2001, but the reporting intensity of charcoal burning suicide surpassed the reporting of suicides using other methods at the end of 2001 and the early 2002. As can be seen from the top two panels of figure 1, the rapid rise in the reporting intensity of charcoal burning suicide coincided with a rapid rise in the incidence of charcoal burning suicides. The peak for the reporting of charcoal burning preceded the peak for the incidence of charcoal burning deaths. However, when the reporting intensity of charcoal burning suicide decreased in early 2002, the number of charcoal burning suicides remained high.

The 3rd and 4th panels of figure 1 plot the derivatives of suicide incidence (panel 3) and news reports per suicide (panel 4). The derivatives indicate the rate of change. When the value is greater than 0, it suggests that the intensity is increasing and when the value is less than 0, it indicates the intensity is decreasing. The period when the reporting of charcoal burning suicide accelerated (2001 to early 2002, when the derivatives are >0) coincided with the time period when charcoal burning suicides began to increase exponentially in Taiwan (panel 3 and panel 4, figure 1). When the reporting intensity of charcoal burning suicide declined in early 2002 (panel 4, figure 1), the incidence of charcoal burning suicide remained at a high level (panel 1, figure 1), although this period coincided with a fall in the rate of increase in charcoal burning deaths (panel 3, figure 1).

As illustrated in the figure (see top panel), the peak of charcoal burning suicide (June 2002) occurred after the peak of reporting of charcoal burning suicide (Feb. 2002), suggesting that news reporting could have contributed to the momentum for the spread of charcoal burning suicide in the community. Furthermore, when the rate of change of charcoal burning suicide increased, the rate of change of other methods declined (panel 3) indicating some possible substitution of methods; similarly, when charcoal burning suicide was excessively reported, the reporting intensity (number of reports per case) of other methods of suicide decreased and vice versa (panel 4). It is important to note that the incidence of charcoal burning remained at a high level even after the reporting intensity has reduced. This suggests that by this stage the method of charcoal burning suicide had penetrated into the community and become self-perpetuating, regardless of further reporting.

Table 1 presents the Poisson regression analysis investigating the impact of news reporting intensity of charcoal and other methods of suicide on the incidence of method-specific suicide rates. The reporting of charcoal burning suicide was positively associated with an increase in the next day occurrence of charcoal burning suicide, each reported news item on charcoal burning suicide was associated with a 16% increase in charcoal burning suicide in the following day (p<.0001) (see footnote under Table 1 for detailed explanations). In contrast, the reporting of other methods of suicide was not related to the occurrence of such suicides on the next day. The association between news reporting and actual suicide did not attenuate when potential confounders were controlled for. Our presentation and the findings of the Poisson regression analysis were restricted to suicides on the following day, whereas we know the effect of any single report is likely to last more than this [16]. Our time series data indicated that altogether, three of the seven p values for lags from day 1 to day 7 for charcoal burning suicide were <0.01 (lag 1, lag 6 and lag 7), whereas the lowest of the seven p-values for the other methods of suicide was 0.41, indicating effects of reporting were not restricted to suicides occurring on the following day.

**Table 1:** The impact of suicide news reporting (charcoal burning and non-charcoal burning respectively) in the previous day on suicide incidence in the following day in Taiwan, 1998–2002.

	Charcoal burning (CB) suicide			
	Unadjusted^a^		Adjusted^b^	
	estimate (s.e.)	p value	estimate (s.e.)	p value
News reporting of CB suicide in the previous day	0.14 (0.03)	<0.0001	0.15 (0.04)	<0.0001
	**Non-Charcoal burning (non-CB) suicide**			
	**Unadjusted^a^**		**Adjusted** c	
	**estimate (s.e.)**	**p value**	**estimate (s.e.)**	**p value**
News reporting of non-CB suicide in the previous day	−0.00 (0.00)	0.74	0.00 (0.00)	0.60

* Note:^ a^Poisson generalized linear autoregressive model controlling for secular trend;

b Poisson generalized linear autoregressive model controlling for secular trend, secular trend of non-charcoal burning suicide count and days of the week;

c Poisson generalized linear autoregressive model controlling for secular trend, secular trend of charcoal burning suicide count and days of the week.

* Note: The Poisson estimates can be transformed to proportions by exponentiation of the estimate and minus 1. For example, in the unadjusted models, the Poisson estimates 0.14 and −0.00 can be transformed to exp (0.14) −1 ≈15% and exp (−0.00) −1≈ 0% respectively. These estimates mean that before adjusting for potential confounders, each news article on charcoal burning suicide was associated with a 15% [95% CI = 0.08, 0.22] increase in charcoal burning suicide on the following day; whereas news reporting of non-charcoal burning suicide was not associated with an increase in non-charcoal burning suicide. After adjusting for potential confounders, for each reported news article on charcoal burning suicide was associated with a 16% (exp (0.15) −1 ≈ 16%) [95%CI = 0.07, 0.26] increase in charcoal burning suicide on the following day.

To assess potential mutual causation (i.e. increased suicide led to increased reporting), Table 2 presents the results of the Poisson regression analysis assessing the influence of charcoal burning and other suicides on newspaper reporting of method-specific suicide rates. The results suggest that the occurrence of both charcoal burning and non-charcoal burning suicides are strongly related to the reporting of method-specific suicide rates on the following day (P<.0001). However, the effect size for charcoal burning deaths was much larger indicating that charcoal burning suicide was considered more ‘newsworthy’. Increase in one case of charcoal burning suicide was associated with an increase of 31% in the rate of news reporting on charcoal burning suicide (P<0.0001); whereas the increase in one case of non-charcoal burning suicide was associated with an increase in 4% of news reporting on non-charcoal burning suicide (see footnote under table 2 for detailed explanations).

**Table 2:** The impact of actual suicides (charcoal burning and non-charcoal burning) on news reporting of charcoal burning and non-charcoal burning suicide in the next day.

	News reporting on charcoal burning (CB) suicide			
	Unadjusted^a^		Adjusted^b^	
	estimate (s.e.)	p value	estimate (s.e.)	p value
CB suicide in the previous day	0.27 (0.04)	<0.0001	0.27 (0.04)	<0.0001
	**News reporting on non-charcoal burning (non-CB) suicide**			
	**Unadjusted^a^**		**Adjusted** c	
	**estimate (s.e.)**	**p value**	**estimate (s.e.)**	**p value**
Non-CB suicide in the previous day	0.04 (0.00)	<0.0001	0.04 (0.00)	<0.0001

CB: charcoal burning, Non-CB: Non-charcoal burning.

* Note: ^a^ Poisson generalized linear autoregressive model controlling for secular trend;

b Poisson generalized linear autoregressive model controlling for secular trend, secular trend of non-charcoal burning suicide count, secular trend of news reporting on non-charcoal burning suicide and days of the week;

c Poisson generalized linear autoregressive model controlling for secular trend, secular trend of charcoal burning suicide count, secular trend of news reporting on charcoal burning suicide and days of the week.

* Note: For each case of charcoal burning suicide was associated with an increase of 31% (exp (0.27) −1≈ 31%) in the rate of news reporting on charcoal burning suicide; whereas the increase in one case of non-charcoal burning suicide was associated with an increase in 4% (exp (0.04) −1 ≈ 4%) of news reporting on non-charcoal burning suicide.

## Discussion

### Main Findings

Newspaper reporting of charcoal burning suicide was associated with an increase in the number of charcoal burning suicides in Taiwan during the early period of the epidemic. Based on our estimation, each reported charcoal burning news item was associated with a 16% increase in next day charcoal burning suicide; whereas the reporting of all other methods of suicide was unrelated to their incidence on the following day. The period when the rate of increase in the newspaper reporting of charcoal burning suicides was at its highest preceded the peak rate for charcoal burning suicide, providing some evidence for a causal effect of news reporting, although other explanations are possible. The reporting intensity of charcoal burning suicide decreased after early 2002 but the incidence of charcoal burning suicide remained high, although the rate of increase of charcoal burning suicides decreased slightly during this period. This finding indicates that when a novel method is made well known, it may become self-sustaining and further reporting may not influence its relative incidence.

### Strengths and Limitations

Our study provides the first empirical evidence of the possible role of newspaper reporting on the charcoal burning suicide epidemic in Taiwan. This is also the first report that illustrates the dynamic and competitive relationships of news coverage for different types of suicide news. However, our study results should be interpreted in light of the following limitations. First, a challenge in the assessment of the association between media reporting and suicide incidence is the problem of reverse causation, i.e. media reporting of suicide events is in part a reflection of its increasing incidence, rather than media ‘causing’ the increase in suicide rates. We assessed the causal direction by exploring the temporal association between media reporting and suicide incidence, i.e. whether rises in reporting preceded rises in suicide and found some evidence that this was the case. Second, suicide rates in Taiwan have been rising since 1993, it is possible that the increasing use of charcoal burning suicide is a reflection of underlying trends in suicide rather than an effect of media reporting, although the rise in charcoal burning deaths occurred 5 years after the more general increase in Taiwan’s suicide rates. Third, ICD codes did not allow us to distinguish charcoal burning suicides from other suicide by gassing, but as the great majority of such suicides were by charcoal burning [9], this will not have a major impact on our analysis. Fourth, suicide news reporting has been measured by counting news items, a content analysis of the articles has not been performed, it is likely that the prominence and content of the articles may influence uptake of the method [17]. However, the quantity of report is in itself an important dimension of media influence on suicide in terms of raising public awareness of a new suicide method. In addition, other types of media channels such as the electronic media and TV stations have not been included in the analysis. Lastly, there are no accurate newspaper circulation data available for all the newspapers in Taiwan; reported sales were based on a telephone survey on a limited sample of Taiwanese. As mentioned earlier, one major newspaper, Liberty Times (LT), was not included to the analysis, as the electronic archives for the newspaper was not available. However, our hand search indicated that LT reported few suicides during the study period, hence may not have affected our results substantially.

### Interpretations and Implications

Although charcoal burning suicide has become one of the most common methods of suicide in some Asian countries [18], it is still not well-understood why the method became so popular in such a short period of time. Our analysis has provided valuable empirical evidence on the possible contribution of extensive newspaper reporting of charcoal burning suicide in the spreading of the novel method. The rapid diffusion of charcoal burning was not associated with reports of a particular celebrity who used this method or other key death, rather, the increase in the quantity of news reporting of the method appeared to be followed by a significant rise in the use of the method. Throughout the study period, charcoal burning suicides were never reported on the front pages of the papers reviewed. Of note, in 1994, before the well-recognized 1998 Hong Kong charcoal burning case, Taiwanese media reported extensively on a suicide pact of two teenage girls who died together by burning barbecue charcoal in a sealed hotel room [19]. The media suggested that they were lesbians who killed themselves because such relationships are not approved by Taiwanese society. Charcoal burning suicide did not spread at that time even though the reporting of the suicide pact persisted for about one week. It is possible that suicide by socially “deviant” figures may be less likely to lead to a modeling effect [17]. Additionally, at that time, the news media focused on the deaths of two smart teenage lesbians rather than the use of a novel method. For example, the headlines of this suicide pact on CT on July 26 1994 read “ Two gifted students from Taipei First Girls High School, Lin and Shi, were found dead on the 25^th^”; on UD the news read “Two students from Taipei First Girls High School left death notes and said goodbye to the world together”. Our finding suggests that cumulative media reporting of a new method adopted by the general population may have the potential to induce a suicide epidemic through a gradual diffusion process.

The finding that after Feb. 2002, the reporting intensity of charcoal burning suicide decreased (second panel, Figure 1) whilst the occurrence of charcoal burning suicide continued to rise (top panel, Figure 1) suggests that the spread of the novel method had become self-sustaining. A likely explanation is that after a while, everyone in a population is aware of the new suicide method (through media exposure, word of mouth and knowledge of friends/family who have used the method). At this point, the media no longer plays a role in increasing awareness of the method. Thus, responsible reporting of novel methods of suicide may be more effective at the very early stage of a potential epidemic.

Our analysis demonstrates the mutual causation between news reporting of charcoal burning suicide and its actual incidence; i.e. the incidence of charcoal burning suicide was associated with its reporting and the reporting of the novel method was related to its future incidence. The process of self-perpetuation through mutual causation may have been a crucial factor for the emergence and the diffusion of the novel method in Taiwan.

Our analysis reveals that reporting of different types of suicide news are competitive. When one type of suicide news gets media attention, other types of suicide news items get less reported.

### Conclusions

This analysis indicates that newspaper reporting may have fuelled Taiwan’s charcoal burning suicide epidemic. Repetitive reporting appears to be harmful, despite the fact that the news items were not placed in the front page, and no celebrity suicides were linked to the use of the method at the early stage of the epidemic. However, once the method had become rooted in the community, the impact of media reporting becomes less prominent; the method takes its own path and becomes self-sustaining. Hence, working proactively with the media to improve the quality of reporting of these tragic deaths and regulate potential harmful reporting are particularly important in the early stage of a suicide epidemic. When a new method becomes widely used, prevention focusing only on regulating media reporting is not adequate. Other intervention measures such as method restriction, gatekeeper training and improve mental health of high risk groups should all be considered.

## References

[1] Chang SS, Gunnell D, Wheeler BW, Yip PSF, Sterne JA (2010) The evolution of the epidemic of charcoal-burning suicide in Taiwan: a spatial and temporal analysis. PLoS Med 7: e1000212. 20052273 10.1371/journal.pmed.1000212 PMC2794367

[2] Chen YY, Yip PSF, Lee C, Fan HF, Fu KW (2010) Economic fluctuations and suicide: a comparison of Taiwan and Hong Kong. Soc Sci Med 71: 2083–2090. 21071128 10.1016/j.socscimed.2010.09.043

[3] Taiwan Suicide Prevention Center (2009) Survey and research. Taiwan Suicide Prevention Center. Available: http://www.tspc.doh.gov.tw/tspc/portal/center/index.jsp. Accessed 2012 October 1.

[4] Yip PSF, Lee DTS (2007) Charcoal-burning suicides and strategies for prevention. Crisis 28 (suppl 1)21–27. 26212191 10.1027/0227-5910.28.S1.21

[5] Tsai CW, Gunnell D, Chou YH, Kuo CJ, Lee MB, et al (2011) Why do people choose charcoal burning as a method of suicide? An interview based study of survivors in Taiwan. J Affect Disord 131: 402–407. 21236495 10.1016/j.jad.2010.12.013

[6] Liu KY, Beautrais A, Caine E, Chan K, Chao A, et al (2007) Charcoal burning suicides in Hong Kong and urban Taiwan: an illustration of the impact of a novel suicide method on overall regional rates. J Epidemiol Community Health 61: 248–253. 17325404 10.1136/jech.2006.048553 PMC2652925

[7] Lin LY (2008) A historical experiment with deregulation: the changes and challenges of the press after the lifting of the press ban in Taiwan. Mass Commun Res 95: 183–212.

[8] Chang SS, Sterne JA, Lu TH, Gunnell D (2010) ‘Hidden’ suicides amongst deaths certified as undetermined intent, accident by pesticide poisoning and accident by suffocation in Taiwan. Soc Psychiatry Psychiatr Epidemiol 45: 143–152. 19363577 10.1007/s00127-009-0049-x

[9] Lin JJ, Chen LH, Huang SM, Lu TH (2008) Problems in estimating the number of suicides by charcoal burning in Taiwan. J Epidemiol Community Health 62: 566. 10.1136/jech.2007.065771 18477760

[10] Chen F, Huggins RM, Yip PSF, Lam KF (2008) Nonparametric estimation of multiplicative counting process intensity functions with an application to the Beijing SARS epidemic. Commun Stat - Theo M 37: 294–306.

[11] Chen F, Yip PSF, Lam KF (2011) On the local polynomial estimators of the counting process intensity function and its derivatives. Scand J Stat 38: 631–649.

[12] Chen F (2011) Maximum local partial likelihood estimators for the counting process intensity function and its derivatives. Stat Sinica 21: 107–128.

[13] Chen YY, Chen F, Yip PSF (2011) The impact of media reporting of suicide on actual suicides in Taiwan, 2002–05. J Epidemiol Community Health 65: 934–940. 21134908 10.1136/jech.2010.117903

[14] Pirkis JE, Burgess PM, Francis C, Blood RW, Jolley DJ (2006) The relationship between media reporting of suicide and actual suicide in Australia. Soc Sci Med 62: 2874–2886. 16387400 10.1016/j.socscimed.2005.11.033

[15] Niederkrotenthaler T, Voracek M, Herberth A, Till B, Strauss M, et al (2010) Role of media reports in completed and prevented suicide: Werther v. Papageno effects. Br J Psychiatry 197: 234–243. 20807970 10.1192/bjp.bp.109.074633

[16] Sisask M, Varnik A (2012) Media roles in suicide prevention: a systematic review. Int J Environ Res Public Health 9: 123–138. 22470283 10.3390/ijerph9010123 PMC3315075

[17] Niederkrotenthaler T, Till B, Kapusta ND, Voracek M, Dervic K, et al (2009) Copycat effects after media reports on suicide: a population-based ecologic study. Soc Sci Med 69: 1085–1090. 19682782 10.1016/j.socscimed.2009.07.041

[18] Wu KCC, Chen YY, Yip PSF (2012) Suicide Methods in Asia: Implications in Suicide Prevention. Int J Environ Res Public Health 9: 1135–1158. 22690187 10.3390/ijerph9041135 PMC3366604

[19] Guan RJ (2010) The aftermath of the suicide of an intelligent young girl from an elite senior high school. PC home Personal News Channel [in Chinese]. Available: http://mypaper.pchome.com.tw/kuan0416/post/1320901966. Accessed 2012 October 1.

